# The University and Hospital Bulletin

**Published:** 1905-07

**Authors:** 


### The University and Hospital Bulletin

DEVOTED TO THE INTERESTS OF THE UNIVERSITY OF BUFFALO AND THE HOSPITALS.

EDITED BY
NELSON W. WILSON, M. D.

*Assistants:*
E. R. McGuire, M. D., Burton T. Simpson, M. D.

Under the auspices of the Faculty of the Medical Department of the University

M. D. Mann, A. M., M. D., Dean,
Charles G. Stockton, M. D.,
John Parmenter, M. D.,
Eli H. Long, M. D.,
Roswell Park, A. M., M. D., LL. D.,
Charles Cary, M. D.,
Herbert M. Hill, A. M., Ph. D.,
Herbert U. Williams, M. D.

AND

*Buffalo General Hospital* — Henry Reed Hopkins, M. D.
*Hospital of the Sisters of Charity* — Eugene A. Smith, M. D.
*German Hospital* — Herman E. Hayd, M. D.

### Alumni Association, Medical Department, U. of B.

The Thirtieth annual meeting of the Alumni Association of the medical department of the University of Buffalo was called to order by the President, Dr. DeLancey Rochester, in the Alumni Hall, at 8.30 p. m., May 3, 1905.

The minutes of the previous meeting were read and approved.

The treasurer's report was received and referred to an auditing committee, which later reported that the accounts and accompanying vouchers had been examined and found correct, with a balance of $160.70 in hand. The report, signed by the committee, William Warren Potter and Allen A. Jones, was filed.

Dr. A. T. Lytle, chairman of the executive committee, reported as follows:

[This report, not having been supplied, must be omitted.—Editor.]

Moved by Dr. Potter and seconded by Dr. A. A. Jones that the report be received and spread upon the minutes; and further, that the thanks of the association be tendered the committee for the able manner in which they had discharged their duties. Carried.

The secretary read an obituary notice of Dr. Ava Melissa Carroll. On motion it was ordered that the report be spread upon the minutes, and a suitable acknowledgment made to Dr. Jane W. Carroll, mother of the deceased.

The President appointed a nominating committee consisting of Irving M. Snow, N. L. Burnham, and B. F. Rogers, which reported nominations as follows: President, Fridolin Thoma, '89, Buffalo; first vice-president, W. R. Campbell, '80, Niagara Falls; second vice-president, M. B. Shaw, '66, Eden Center; third vice-president, Mary M. Huntley, '96, Buffalo; fourth vice-president, G. A. Himmelsbach, '91, Buffalo; fifth vice-president, H. P. Trull, '67, Williamsville; treasurer, H. K. DeGroat; secretary, N. G. Russell.

*Executive Committee.*—A. T. Lytle, chairman; Franklin W. Barrows, G. W. Wende; *ex-officio,* F. Thoma, E. H. Long.

*Trustees.*—A. W. Hènchell, Rochester; A. W. Bayliss, Buffalo; R. P. Bush, Horseheads; D. W. Harrington, Buffalo; William M. Ward, North Collins.

On motion the secretary was ordered to cast one ballot, which was done, and the nominees were declared duly elected.

The president delivered his annual address (see p. 701 June issue of the JOURNAL) and was followed by Dr. E. L. Shurly, of Detroit, with a paper, entitled, "Concerning the Etiology of Appendicitis. (See page 773, this issue.)

The discussion was opened by Dr. Eugene A. Smith.

Dr. M. D. MANN presented a verbal report on general matters of interest relating to college affairs, announcing among other things that the term would be lengthened one month, and the minimum marking increased to 75.

Moved by Dr. W. W. POTTER and seconded by Dr. A. A. Jones that the thanks of the association be tendered the faculty in consideration of raising the standard and lengthening the course of study.

On motion the thanks of the association were tendered Dr. Rochester and Dr. Shurly for the excellent papers presented.

The meeting adjourned without day, and the alumni proceeded to the library where they were the guests of the faculty at a smoker.

